# Genetic testing enhances diagnosis in critically ill neonates: insights from the first Colombian cohort

**DOI:** 10.3389/fped.2025.1605166

**Published:** 2025-08-21

**Authors:** Paula Rueda-Gaitán, Diego Alejandro Rodríguez Gutiérrez, Yuri Natalia Sanchez Rubio, Yina D. Carrillo, Leslie Ivonne Martínez de la Barrera, Néstor Nenroth Muñetones Reina, Mario Isaza-Ruget, Juan Javier López Rivera

**Affiliations:** ^1^ Laboratorio Clínico Especializado, Clínica Universitaria Colombia, Clínica Colsanitas, Bogotá, Colombia; ^2^ Fundación Universitaria Sanitas, Unidad de Investigaciones, Grupo de Investigación INPAC, Bogotá, Colombia; ^3^ Unidad de Neonatología, Clínica Universitaria Colombia, Clínica Colsanitas, Bogotá, Colombia; ^4^ Unidad de Neonatología, Clínica Reina Sofía, Clínica Colsanitas, Bogotá, Colombia; ^5^ Grupo de Genética Médica, Clínica Universitaria Colombia, Clínica Colsanitas, Bogotá, Colombia

**Keywords:** NICU (neonatal intensive care unit), genomics, WES (whole exome sequencing), aCGH, precision medicine & genomics

## Abstract

**Introduction:**

The integration of genetic testing in pediatrics has advanced significantly in recent years. The incorporation of technologies such as Next Generation Sequencing (NGS) and array-based Comparative Genomic Hybridization (aCGH) in increasingly younger patients has accelerated the transition toward precision medicine.

**Methods:**

This retrospective cross-sectional study (January 2021–June 2024) included 187 neonates (≤90 days old) from the NICUs of the Clínica Colsanitas network in Bogotá, Colombia and evaluate the diagnostic yield for genomic testing comprising 82 Whole Exome Sequencing (WES) and 125 aCGH tests, with 18 patients undergoing both. This study also examined the phenotypic traits of patients to investigate potential associations with a higher diagnostic yield. Symptoms were characterized using Human Phenotype Ontology (HPO) terms and analyzed with a propagation algorithm for improved accuracy.

**Results:**

The diagnostic yield was 30.5% for WES and 8% for aCGH. Noteworthy, we identify four novel SNVs with potential pathogenicity and report a rare case of co-occurring deletion and duplication, both previously unreported in the literature. Phenotypic analysis revealed a strong association between what were considered “growth abnormalities” related to intrauterine growth restriction, low birth weight, and/or growth retardation, with “head or neck abnormalities” related to specific malformations of the face or head and/or dysmorphic facial phenotypes.

**Discussion:**

These findings highlight the importance of applying, in particular, WES as a first-level clinical diagnostic test in patients with suspected genetic or complex diseases who are hospitalized in the NICU. Consequently, it is hoped that these results will support the development of clinical guidelines for the integration of molecular genetic testing into neonatal care in Colombia.

## 1 Introduction

Neonatal infections, prematurity, and congenital anomalies remain the primary causes of neonatal mortality, with the latter two often linked to potential genetic disorders (1). It is estimated that nearly half of congenital anomalies result from single-gene variants (2). Consequently, international guidelines have been established to recommend the use of advanced molecular testing, such as whole exome sequencing (WES), whole genome sequencing (WGS), and array-based comparative genomic hybridization (aCGH), for neonates admitted to neonatal intensive care units (NICUs) (3, 4). The effectiveness of these diagnostic approaches relies heavily on appropriate patient selection, adherence to clinical practice guidelines, and the integration of Human Phenotype Ontology (HPO) terms in bioinformatic analysis. Adopting standardized clinical protocols for patient referral ensures efficient resource utilization, expedites accurate diagnoses, and facilitates timely therapeutic interventions, with reported diagnostic yields ranging from 30% to 69% (5–9).

Incorporating molecular genetic testing into neonatal clinical practice is a pivotal step toward precision medicine, enabling healthcare providers to tailor interventions, implement targeted therapies, and guide family decision-making, including reproductive planning (10). This approach not only reduces the diagnostic odyssey and unnecessary invasive procedures but also optimizes healthcare resource allocation. In Colombia, genetic testing has increased in recent years (11, 12), overcoming the social and economic barriers of a middle-income and developing country, and has begun to be used in various populations, including newborns. While the Colombian healthcare system covers the costs of molecular tests, including aCGH, WES, and WGS, there are currently no national clinical guidelines to regulate their use, nor studies assessing their diagnostic performance in this population. Therefore, the aim of this study is to describe the first cohort of NICU patients who underwent molecular genetic testing, evaluate the diagnostic yield of WES and aCGH and to investigate whether there are phenotypic characteristics associated with a higher positivity rate, with the ultimate goal of contributing to the evidence base for the development of clinical guidelines for neonatal patients.

## 2 Materials and methods

### 2.1 Study design

This was a retrospective cross-sectional study carried out from the information system of the Clinical Laboratory of Clínica Colsanitas in the period from January 2021 to June 2024. All patients were assessed by a multidisciplinary team of physicians, including a clinical geneticist, who selected the appropriate diagnostic tests (WES, aCGH) based on clinical criteria. Patients for whom maternal and paternal samples were available at the time of medical care for the test were studied in trios; otherwise, individual studies were conducted. Patients were included if they fulfilled the following criteria:

• Patients younger than 90 days admitted to the NICUs of the Clinica Colsanitas network. In preterm patients, age correction based on gestational age was applied only when it resulted in a positive corrected age, as negative values are not clinically meaningful.
• Patients in whom an aCGH or WES (individual or trio) was requested during their stay in the NICU.

Patients were also excluded if:

• The genomic tests request was made because of a family history of genomic alterations unrelated to their phenotype.
• Patients with a clear suspicion of aneuploidy or with a karyotype result of trisomy 13, 18 or 21.

Preterm infants, including those with complications of prematurity, were not excluded from the cohort, as complex presentations in this subgroup often raise suspicion for an underlying genetic etiology.

The parents of the patients signed the informed consent form prepared by the Clinical Laboratory of Clínica Colsanitas for the performance of genetic tests and the use of anonymized data for research. All informed consent forms for molecular studies conducted at Clínica Colsanitas include a list of items that parents must sign “yes” or “no” for, including the following: “(1) I have received information about the indication, purpose, and risks of this genetic study.” “(2) I have read and understood the information about the genetic study, its limitations, and its possible results.” “(3) I understand that the data obtained may help in the management of the disease under study, and I give my consent for the information to be used by the specialized laboratory at Colsanitas Clinic for research purposes, publication in databases, and audits, as this information will be anonymous in any of these cases.” For the cases described here, all parents accepted these points.This study was submitted and approved by the Research Ethics Committee of Clínica Colsanitas.

In addition, all patients received both pre-test and post-test genetic counseling. For post-test counseling, the NICU follows a protocol whereby all inpatients are counseled at the time the genetic result becomes available. If the patient has been discharged by the time the results are issued, a priority care pathway is activated: the pediatrician promptly evaluates the patient and initiates an expedited referral to clinical genetics to ensure timely post-test counseling.

### 2.2 Genomic tests

#### 2.2.1 Whole exome sequencing

Exome sequencing was performed using the Twist Comprehensive Exome v1 Kit. Following genomic DNA (gDNA) extraction and concentration normalization, tagmentation, amplification, and ligation of unique sample markers were carried out. Libraries were enriched via hybridization with biotinylated probes targeting exonic regions, followed by final amplification, purification, and quality assessment, requiring a concentration above 3 ng/µl and a fragment size of ∼330 bp. Sequencing was conducted on the NovaSeq6000 (Illumina), and data were processed using Varsome Clinical with the GRCh37/Hg19 reference genome. Variant interpretation followed ACMG guidelines (13) and ClinGen recommendations. The minimum quality metrics for case analysis were 95% total coverage and more than 100× depth. In addition, all identified variants had at least 50 reads depth and an allele fraction greater than 30%.

#### 2.2.2 aCGH

For aCGH processing, genomic DNA was first extracted from a peripheral blood sample using the protocol suggested by the manufacturer company, Agilent®. DNA digestion was performed using Alu I and RSA I enzymes from both patient and reference samples, followed by fluorochrome labelling of patient DNA and reference DNA (male and female control) using Cy5 and Cy3 fluorochromes. The DNA was then purified on columns and quantified to ensure concentration, yield and specific activity, parameters necessary to compare patient and reference DNA. Hybridisation was then performed using the Agilent® SurePrint G3 Human ICGH+SNP 4 × 180 K array. Finally, the scan was performed using the SureScan® platform and data was acquired, quality parameters were evaluated and results were analyzed using Agilent CytoGenomics v5R software. Analysis was performed using available databases: DECIPHER, DGV (Database of Genomic Variants), ClinVar-National Center for Biotechnology and the American College of Genetic Medicine guidelines for interpretation and reporting of copy number variants (14).

### 2.3 Data analysis and interpretation

#### 2.3.1 Statistical descriptive analysis

A statistical descriptive analysis was performed to determine the number of patients who underwent at least one of the two types of molecular genetic testing: WES (single and trio) and aCGH. Patients were stratified according to sex, and it was determined how many male and female patients were referred for each of these tests. In addition, the results of each test were classified into three categories: positive, negative, and variants of uncertain significance (VUS).The overall diagnostic yield of the genetic tests was determined by considering the proportion of positive results (those with pathogenic or likely pathogenic variants explaining the patient's phenotype) compared to VUS or negative results.

A variant statistical analysis was also performed including all variants (SNVs, deletions, insertions and CNVs) detected with both tests. This analysis allowed us to identify those variants that occurred more frequently in the population with positive results, novel variants that have not been reported in the databases and the chromosomes and the most frequently altered chromosomes.

On the other hand, due to the sample size and the phenotypic heterogeneity of the patients, Fisher's exact test was used to statistically assess whether there was a significant association between the presence of specific phenotypes and the results of the molecular studies (15). This was done by analyzing the proportion of the event in the group of interest. For this calculation, only cases with a pathogenic or likely pathogenic variant explaining the patient's phenotype were considered positive. Reports containing variants of uncertain significance (VUS) or no clinically significant variants were classified as negative. The formula is defined as follows:

$$p=\frac{\left(\frac{a+c}{a}\right)\left(\frac{b+d}{b}\right)}{\left(\frac{n}{a+b}\right)}.$$

To evaluate the association of the positive results vs. the presence or absence of the phenotypes, odds ratios (OR) with their respective 95% confidence intervals were calculated from the following formula:

$$\mathrm{OR}=\frac{\left(a/c\right)}{\left(b/d\right)}$$

$$95\text{\%}\;\mathrm{CI}=e^{\mathrm{Ln}(\mathrm{OR})\pm 1.96\times \sqrt{\left(1/a)+(1/b)+(1/c)+(1/d\right)}}.$$

#### 2.3.2 Phenotypic analysis

A phenotypic analysis was performed by extracting the most specific HPO terms from the electronic health record (EHR) of each patient. These specific terms were propagated to the highest possible level term within the “Phenotypic abnormality” term hierarchy of 23 terms according to the method described by Galer et al. (16) in order to group similar phenotypes into the same HPO term, reduce the number of HPO terms to be analyzed, and more clearly visualize the patterns present among patients. In this way, a patient with neurodevelopmental delay (HP:0012758) and a patient with intellectual disability (HP:0001249) would be grouped with the HPO “Abnormality of the nervous system” (HP:0000707). The terms “Abnormality of limbs” (HP:0040064) and “Abnormality of the musculoskeletal system” (HP:0033127) are at the same hierarchical level within “Phenotypic abnormality”, and in turn, share terms within their hierarchies. Therefore, a panel of experts determined under which of the two terms the shared phenotypes should be classified. For example, the term Short metacarpal (HP:0010049) was propagated to “Abnormality of limbs” (HP:0040064).

HPO terms propagated were organized in a heatmap according to their similarity, together with the genetic test results of the patients. Dendrograms for both HPO terms and patients were used to identify clustering patterns. The analysis allowed detection of HPO terms with the highest prevalence of positive results, as well as common phenotypic features among patients with pathogenic results and variants of uncertain significance. These dendrograms were used for the construction of the heatmap; a hierarchical clustering analysis was performed to identify phenotypic patterns and determine similarities among patients based on HPO terms. Clustering techniques were used to group patients and phenotypes with similar characteristics, thereby facilitating the identification of diagnostic patterns. To measure the similarity between patient profiles, Euclidean distance was employed. Additionally, Ward's method, which minimizes variance within each group, was applied to optimize the clustering of patients based on their phenotypic characteristics. The graphical representation allowed the identification of groups of phenotypes and their correlation with the different diagnostic results.

## 3 Results

### 3.1 Individuals studied and demographics

**Figure 1:**
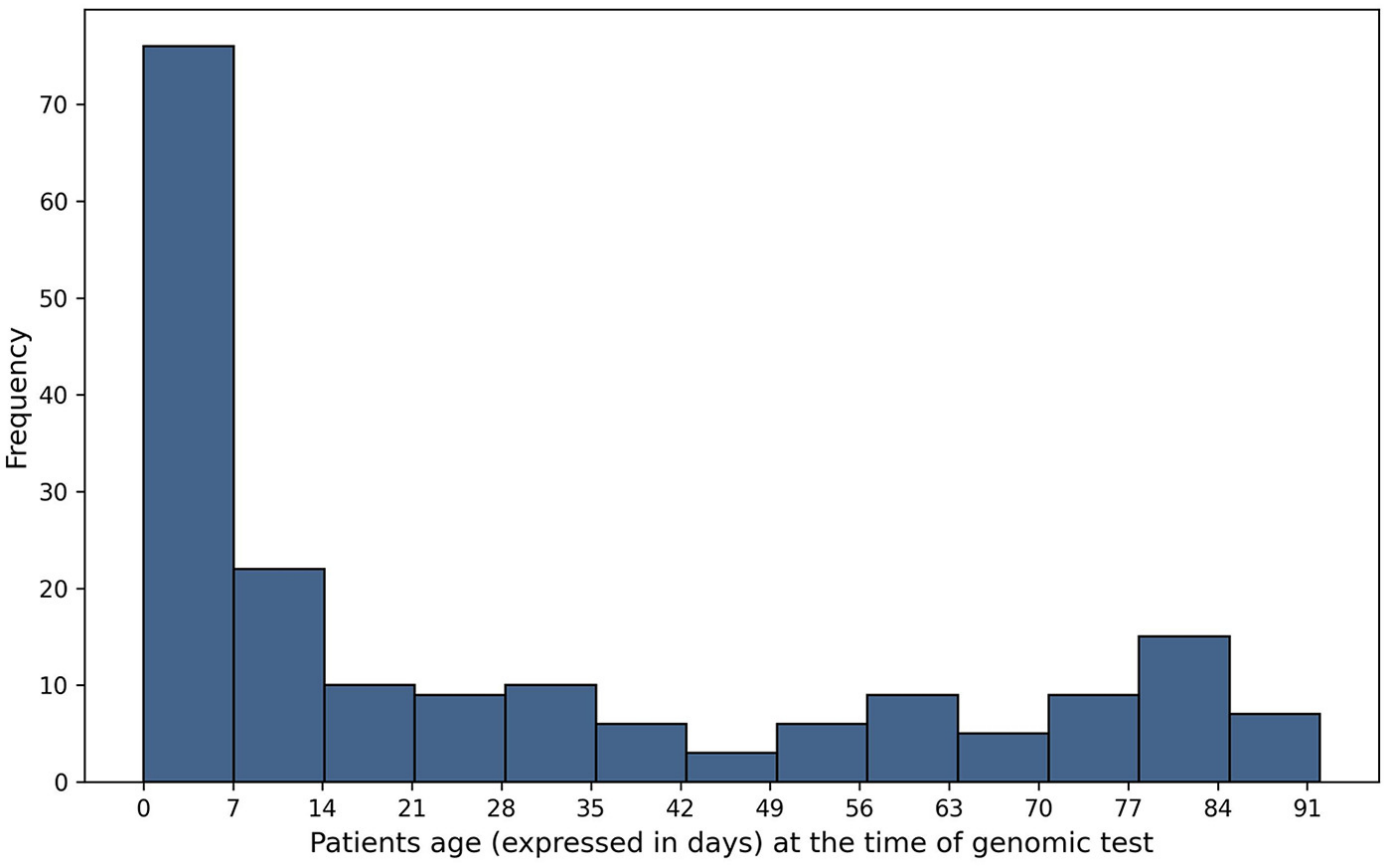
Age distribution of patients at the moment of the test.

A cohort of 187 patients less than 90 days old was evaluated. The median age of the patients at the time of genomic test ordering was 13 days (IQR: 51 days, range: 90 days). 47.5% (*n* = 89) were male and 52.4% (*n* = 98) were female (Figure 1). A subset of 18 patients underwent simultaneous WES and aCGH testing, resulting in a total of 205 diagnostic tests. In total, 250 samples were processed, as WES included both single and trio testing.

Given the limitations of the sample size, patients were classified according to their clinical indication to estimate the diagnostic yield. The distribution of patients by clinical indication is described in Table 1.

**Table 1:** Frequency of patients according to clinical indication.

Clinical indication	*n*	%	Clinical indication	*n*	%
Multiple congenital malformations	30	16.0	Respiratory distress syndrome	3	1.6
Facial dysmorphism	27	14.4	Family history	2	1.1
Cardiopathy	15	8.0	Paralysis	2	1.1
Skeletal malformations	13	7.0	Neonatal jaundice	2	1.1
Growth disorders	14	7.5	Hypothyroidism	2	1.1
Neurological disorder	11	5.9	Ocular anomaly	2	1.1
Brain malformations	8	4.3	Metabolic disorder	2	1.1
Neural tube defects	7	3.7	Swallowing disorder	1	0.5
Not indicated	7	3.7	Syndactyly	1	0.5
Abdominal wall defect	6	3.2	Microcephaly	1	0.5
Digestive system malformations	5	2.7	Kidney malformations	1	0.5
Hypotonia	5	2.7	Respiratory tract malformation	1	0.5
Genitourinary malformations	4	2.1	Bile duct malformation	1	0.5
Congenital anomalies of the urinary tract	4	2.1	Hydrops fetalis	1	0.5
Epileptic syndrome	5	2.7	Hydrocephalus	1	0.5
Dermatological disorder	3	1.6			
Total	**187**				

### 3.2 Identified variants

#### 3.2.1 WES results

A total of 82 WES tests were performed, with 46.3% (*n* = 38) conducted on male patients and 53.7% (*n* = 44) on female patients. The tests included 60 single exomes (73.2%) and 22 trios (26.8%). Of these, 35 (42.7%) yielded negative results, while 25 (30.5%) were positive (*n* = 15 single; *n* = 10 trio), confirming pathogenic genetic variants linked to the investigated diseases. Additionally, 22 tests (26.8%) identified variants of uncertain significance (VUS).

Among the positive WES, 5 patients had one or more CNVs, with one patient having both a deletion and a duplication, identifying a total of 6 CNVs (*n* = 6). 5 CNVs were classified as pathogenic (*n* = 5) (3 duplications and 2 deletions) and one was likely pathogenic (*n* = 1) (1 deletion).

Of the 20 SNVs reported in WES, 4 were found that had not been reported in Clinvar or other databases, the description of the novel variants reported is shown in Table 2.

**Table 2:** Description of the novel variants reported in exomes.

Patient	Gene	Variant nomenclature	Classification	ACMG Criteria	Transcript	OMIM-related condition
P1	*GRIN1*	c.1824G>C p.Trp608Cys	P	PS2, PM1, PM2, PP2, PP3	NM_007327.3	Neurodevelopmental disorder with or without hyperkinetic movements and seizures, autosomal dominant (AD).
						Neurodevelopmental disorder with or without hyperkinetic movements and seizures, autosomal recessive (AD).
						Developmental and epileptic encephalopathy 101 (AR).
P2	*ABCA12* *	c.1789del p.Ser597Leufs*18	LP	PVS1, PM2	NM_173076.3	Ichthyosis congenital autosomal recessive 4A (AR). Ichthyosis congenital autosomal recessive 4B (harlequin) (AR).
P3	*GDF1*	c.885C>A p.Tyr295*	LP	PVS1, PM2	NM_001492.6	Congenital heart defects multiple types 6 (AD). Right atrial isomerism (Ivemark) (AR).
P4	*FBN1*	c.7704_7705insTGTG p.Asp2569Cysfs*5	LP	PVS1, PM2	NM_000138.5	Acromicric dysplasia (AD). Ectopia lentis familial (AD). Geleophysic dysplasia 2 (AD). Marfan lipodystrophy syndrome (AD). Marfan syndrome (AD). MASS syndrome (AD). Stiff skin syndrome (AD). Weill—Marchesani syndrome 2 dominant (AD).

* This variant was found in compound heterozygosis.

**Figure 2:**
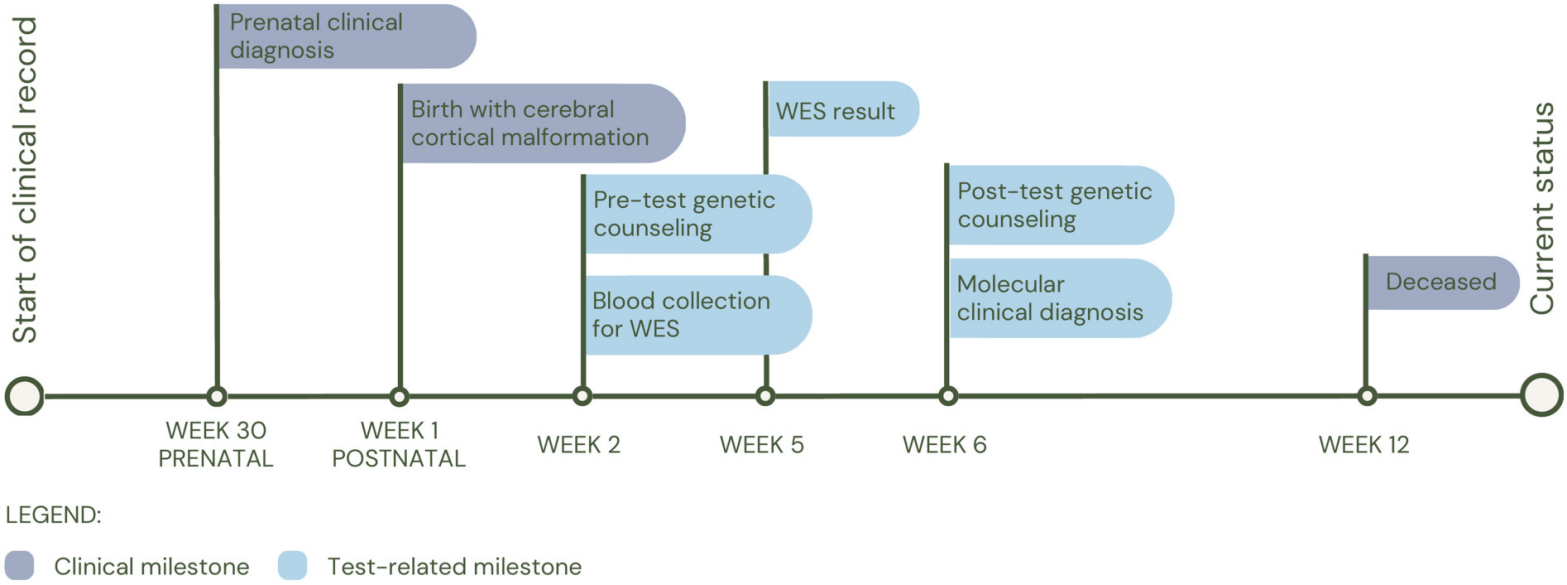
Diagnostic timeline for P1.

The first patient (P1) was a 14-day-old female with prenatal diagnosis of cerebral cortical malformation, polymicrogyria, colpocephaly and secondary ventriculomegaly, identified by cerebral MRI at 30 weeks of gestation. Five weeks after birth, a trio of WES was performed and a *de novo* heterozygous variant in the *GRIN1* gene (c.1824G>C; p.Trp608Cys) associated with neurodevelopmental disorder with an autosomal dominant inheritance pattern with or without hyperkinetic movements and seizures (MIM#614254) was identified. It has been shown that patients affected by variants in the gene, in addition to seizures and neurodevelopmental disorder, have alterations in radial and tangential neuronal migration (17). The patient had a fatal outcome at three months of age (See Figure 2 for diagnostic timeline).

**Figure 3:**
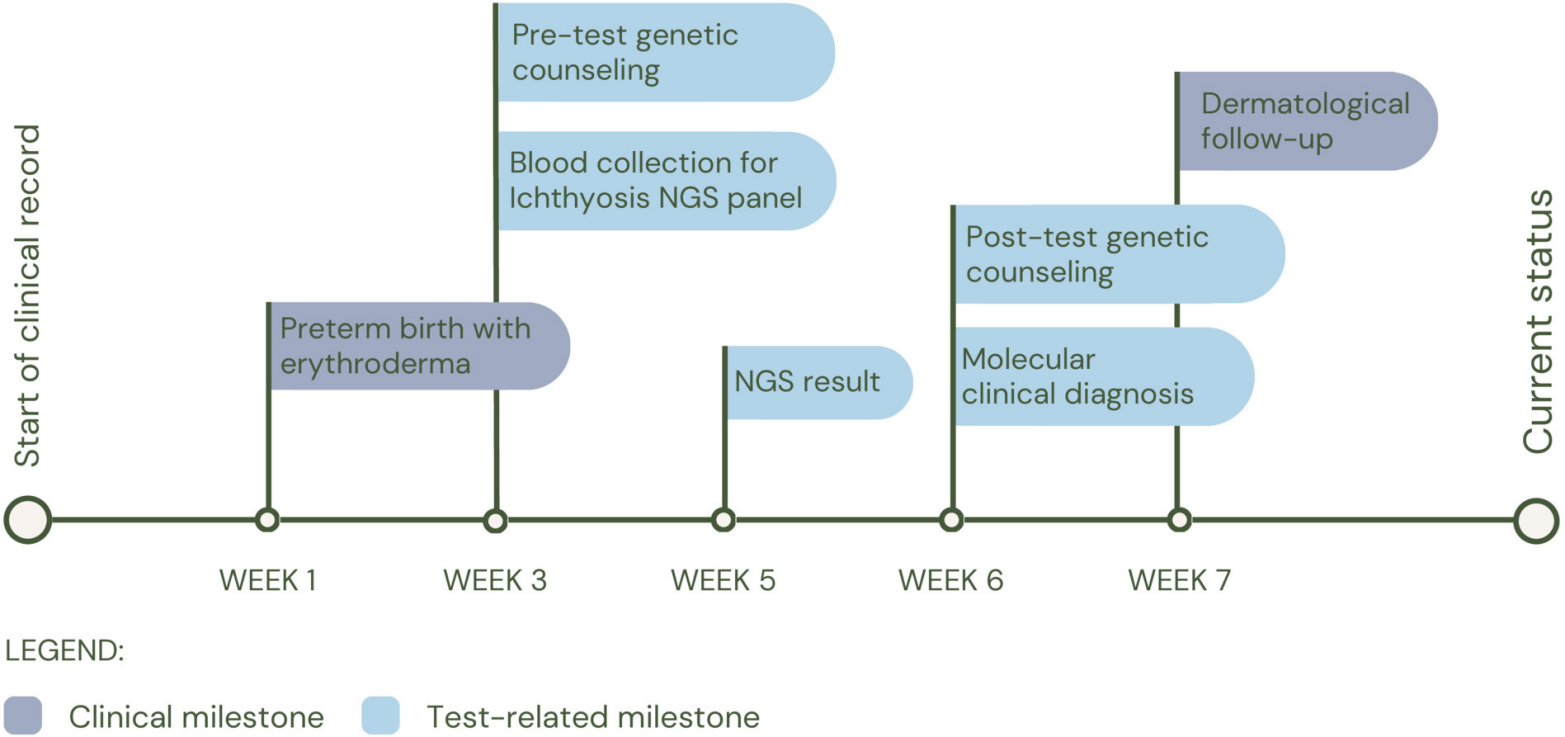
Diagnostic timeline for P2.

Patient 2 (P2) was born preterm at 33 weeks of gestation and 25 days old at the time of the study request, with a pediatric dermatology diagnosis at birth of congenital ichthyosiform erythroderma, retrognathia, macroglossia and glossoptosis, who underwent an individual WES 3 weeks after birth. The study identified a possible compound heterozygosity in the *ABCA12* gene, consisting of the variants c.4139A>G (p.Asn1380Ser), reported as pathogenic in the Clinvar database (rs28940269) and c.1789del (p.Ser597Leufs*18), which has not been reported in the databases so far. The NMD prediction tool classifies this variant as subject to nonsense-mediated decay degradation. Currently, it has not been possible to study the parents to evaluate the segregation of the variants in each parent, which would allow us to confirm the diagnosis of autosomal recessive congenital ichthyosis 4A (MIM#601277) or autosomal recessive congenital ichthyosis 4B (harlequin) (MIM#242500). Clinically, the patient was managed under the guidance of the dermatology service, and his treatment regimen consists of intensive moisturization with topical emollients, including Cetaphil® and a therapeutic shower oil (See Figure 3 for diagnostic timeline).

**Figure 4:**
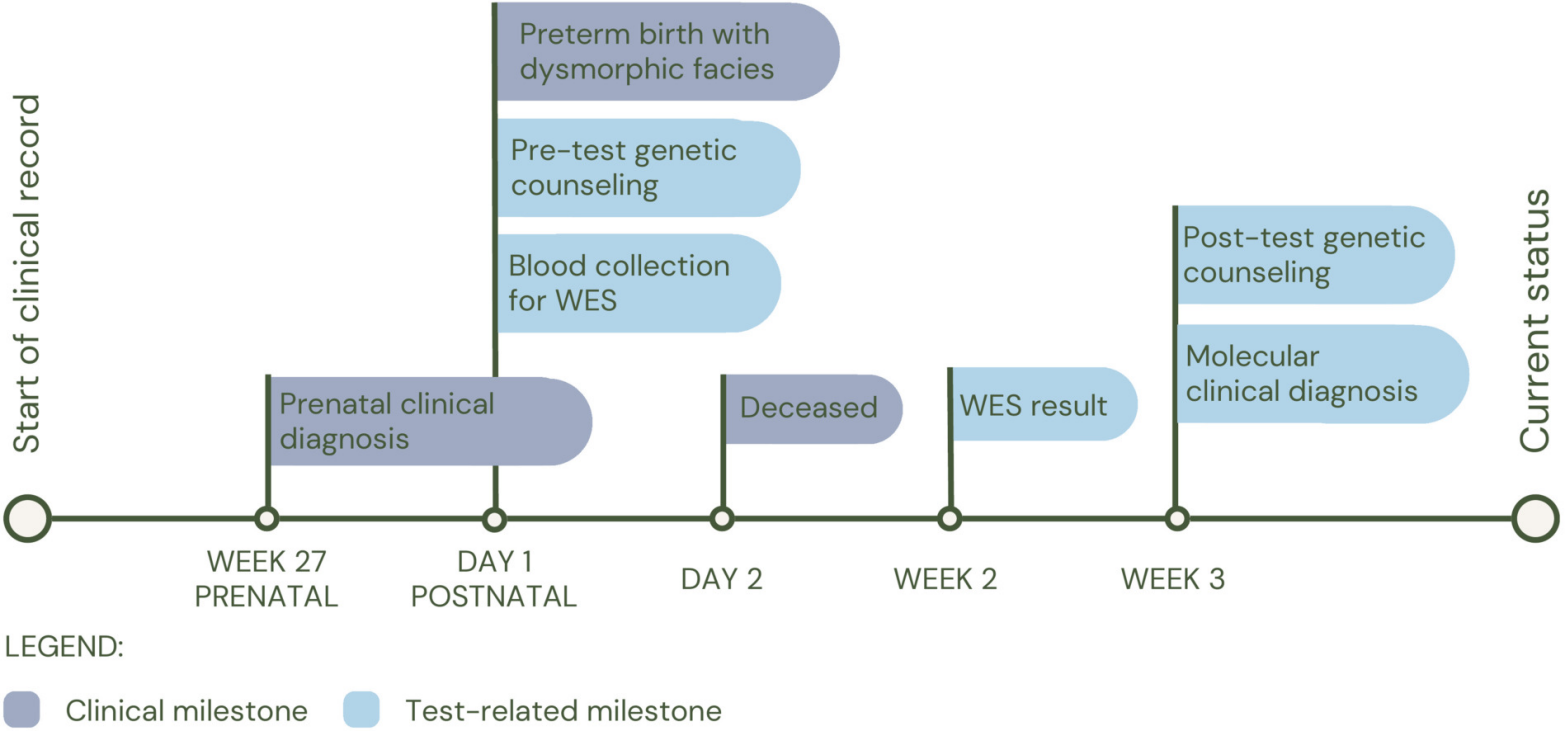
Diagnostic timeline for P3.

The third patient (P3), was born preterm at 30 weeks of gestation, whose clinical picture consisted of dilated cardiomyopathy, ventricular septal defect, incomplete atrioventricular canal, left pulmonary agenesis, macroglossia with glossoptosis, and cavum pellucidum septum. At birth, individual WES was performed and a heterozygous variant was identified, probably pathogenic in the *GDF1* gene (c.885C>A; p.Tyr295*), associated with multiple types of congenital heart disease (MIM#613854). The patient had a fatal outcome at two days of age (See Figure 4 for diagnostic timeline).

**Figure 5:**
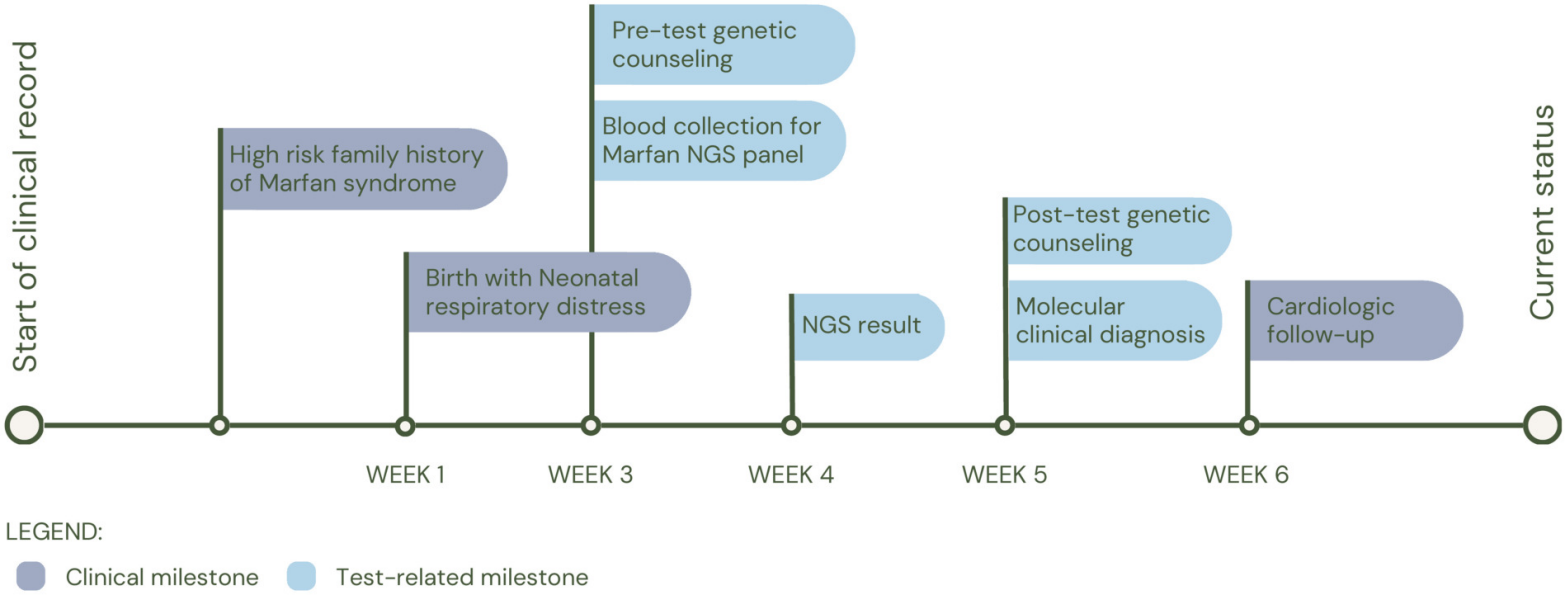
Diagnostic timeline for P4.

Finally, patient 4 (P4), born on the day the test was requested, was referred due to respiratory distress at birth and a history of maternal death during childbirth due to nonmolecularly confirmed Marfan syndrome. It was inferred that the mother shared the *FBN1* variant c.7704_7705insTGTG (p.Asp2569Cysfs*5) with her daughter, in whom it was identified and reported as likely pathogenic. The patient was clinically diagnosed with Marfan Syndrome (MIM#154700). Following the diagnosis, the patient was placed under the care of the cardiology service for periodic follow-up (See Figure 5 for diagnostic timeline).

Furthermore, we would like to emphasize a particular case, as it is the first patient affected with this condition in a cohort of more than 10,000 patients of all ages from the Specialized Laboratory of Clínica Colsanitas. He was a 78-day-old male patient with very low birth weight, severe respiratory symptoms, hyperlactatemia, profound hypoacusis in the right ear and severe hypoacusis in the left ear. He also had a prenatal history of severe intrauterine growth restriction (IUGR) and cytomegalovirus infection. In this patient, the homozygous variant *TRMT10C* c.542G>T (p.Arg181Leu) was identified, which is associated with combined oxidative phosphorylation deficiency 30, an ultra-rare systemic mitochondrial disease. Metodiev et al. (18) suggested through functional studies that this variant affects MRPP1 protein stability and mtRNA processing, without affecting m1R9 methyltransferase activity. Interestingly, the aCGH results in this patient identified loss of heterozygosity (LOH) greater than 5 Mb in 6 regions, including the 3q11.2q12.3 loci, where the *TRMT10C* gene is located.

Among the most frequently reported genes in exome sequencing, TPO was identified in two patients with compound heterozygosity, both presenting with hypothyroidism as their primary clinical concern. Pathogenic variants in TPO cause a severe form of congenital hypothyroidism, characterized by the immediate release of accumulated radioiodide following sodium perchlorate administration (OMIM #606765) (19). The European Society for Paediatric Endocrinology recommends genetic investigation of syndromic congenital hypothyroidism to identify novel genes and facilitate genetic counseling (20). In such cases, trio-based WES proves to be a valuable diagnostic tool, enabling early intervention and informed genetic counseling.

#### 3.2.2 aCGH results

On the other hand, of the 125 aCGH tests performed, 103 were negative (no clinically significant CNV were identified), 10 were positive, and 12 revealed a CNV classified as VUS. In addition, among the cases without CNVs, 25 had LOH greater than 5 Mb, and in 4 of these cases WES was requested due to suspicion of a recessive hereditary disorder. The diagnostic yield of aCGH was 8%. Among the reported CNVs, 67% (*n* = 19) were duplications, while 32% (*n* = 9) were deletions. CNVs affected almost all chromosomes (1, 4, 5, 7, 8, 9, 11, 13, 15, 17, 20, 21, 22, X, and Y), with a higher frequency for chromosomes 17, X, and 21, as expected. Two of the patients who were detected CNV by WES underwent aCGH to confirm the finding.

One of the most frequent CNVs identified was chromosome 21 duplications (*n* = 3), which are associated with Down syndrome, included in the most common chromosomal aneuploidies (21). Affected patients presented with skeletal malformations, facial dysmorphism, and growth disorders. Trisomy 21 was clinically suspected in only one case, while the other two had not yet developed the characteristic phenotype, making diagnosis more challenging. Among these cases, two exhibited a partial duplication affecting only the q arm, while the third had a complete duplication of chromosome 21.

**Figure 6:**
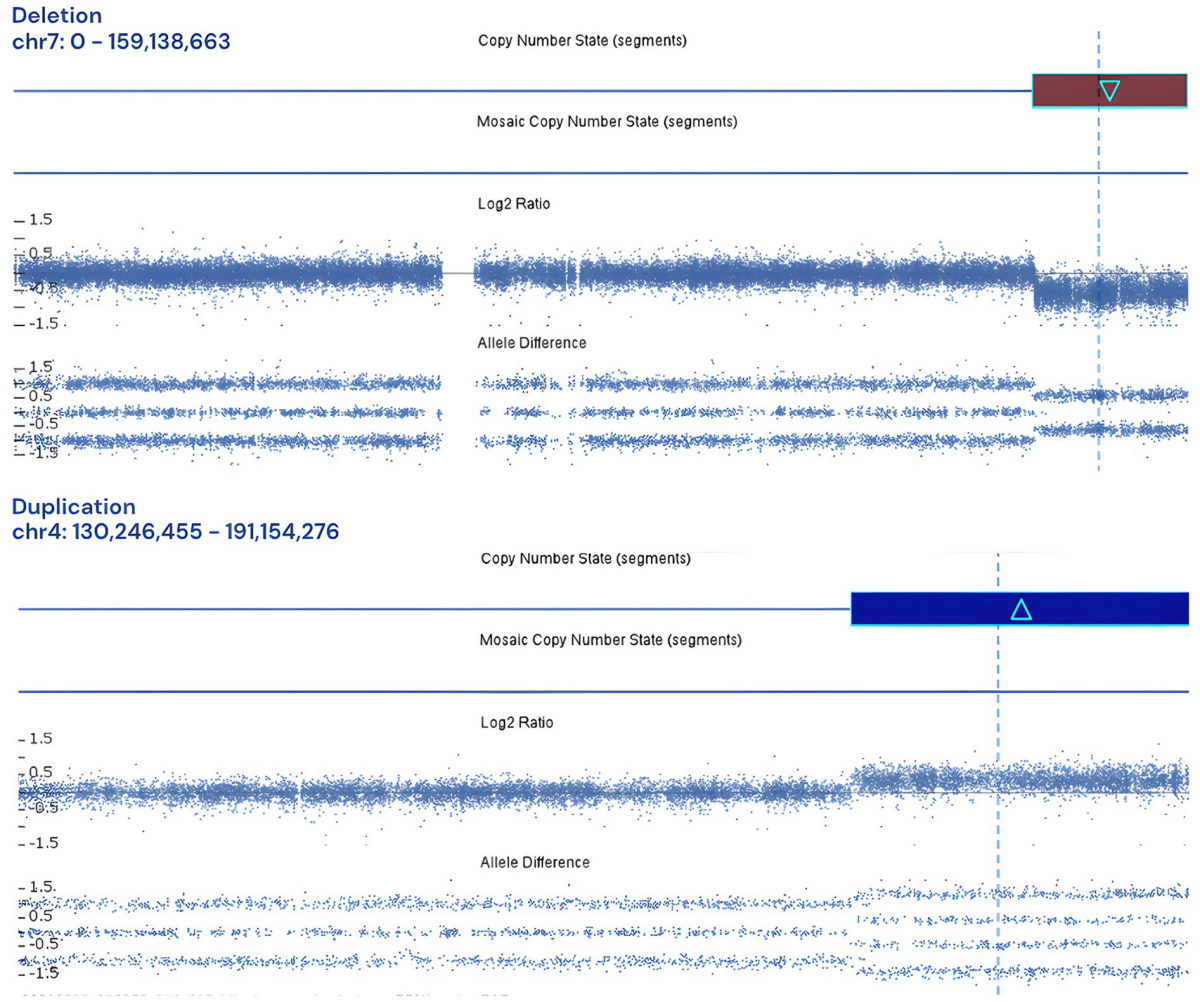
Log2 plot for 17.483 Mb gain on chromosome 4q34.1q35.2 and a 20.839 Mb loss on chromosome 7q34q36.3.

Furthermore, aCGH facilitated the identification of three patients exhibiting concurrent chromosomal loss and gain. The first case involved a five-day-old infant whose prenatal karyotyping at 22 weeks of gestation, conducted due to ultrasound findings of type III cleft palate and intrauterine growth restriction (IUGR), yielded normal results. Nevertheless, aCGH analysis revealed a 17.483 Mb gain on chromosome 4q34.1q35.2 and a 20.839 Mb loss on chromosome 7q34q36.3 (Figure 6), suggesting a balanced chromosomal rearrangement inherited from the father, as maternal karyotyping was normal. Notably, a similar chromosomal profile was reported in 2018, associated with single ventricle anomalies, partial thalamic fusion, and polycystic kidneys (22).

The second patient presented a loss of 15.4 Mb on 9p24.3p22.3 and a gain of 25.3 Mb on chromosome 11q22.3q25. Duplications in the 11q22.3q25 region have been reported in the literature in the context of translocations with other regions, associated with variable phenotypes such as dysmorphic facial features, minor cardiac anomalies, central nervous system anomalies and psychomotor retardation. For instance, Lekszas et al. (23), reported such an alteration by an unbalanced segregation of a paternal t(9;11)(p24.3;p15.4) translocation.

Finally, we identified a patient with a loss of 9.25 Mb on 1p36.33p36.22 and a gain of 31.5 Mb on 1q41q44. To our knowledge, no similar case reports have been published to date. The patient had prenatal findings of total agenesis of the corpus callosum, impaired neuronal migration and colpocephaly. The patient died three days after birth.

Among the 22 patients with CNV classified as pathogenic or VUS, clinical indications included: Multiple congenital malformations (*n* = 2), Genitourinary malformations (*n* = 2), Facial dysmorphism (*n* = 5), Growth disorders (*n* = 3), and Neurological disorder (*n* = 3). In particular, patients with facial dysmorphia had the highest number of reported pathogenic CNVs.

### 3.3 Diagnostic yield and phenotype-based analysis

To summarize, 82 exomes and 125 aCGH tests were performed, 19 patients underwent both tests. The diagnostic yield, calculated as the percentage of positive results for exome, was 30.5% (18.3% for single exomes and 12.2% for trio) and for aCGH was 8%.

To determine whether there was an association between the phenotypes presented by the patients and the outcome of the molecular studies, the individual diagnostic yield and Fisher's exact test were calculated. Categories were defined using the previously described propagation model, resulting in 17 categories based on the HPO hierarchy. For example, the category “Abnormality of head and neck” included the terms “Abnormality of cranial sutures” and “Cleft palate”, which includes structural level anomalies of the head, it is important to note that specific anomalies of the eye and ear are in a different category, these include phenotypes such as Ptosis palpebralis and hearing impairment, respectively. The results of both metrics are shown in Table 3.

**Table 3:** Diagnostic yield and *p*-value Fisher's exact test results for each phenotype category, where *n* refers to the amount of patients with the phenotype.

Phenotype	*n*	Diagnostic yield (%)	*p*-value Fisher's exact test
Growth abnormality	46	23.9	0.384
Abnormality of head or neck	52	25.0	0.212
Abnormality of the cardiovascular system	29	13.8	0.606
Abnormality of prenatal development or birth	20	30.0	0.225
Abnormality of the musculoskeletal system	41	22.0	0.651
Abnormality of limbs	13	7.7	0.467
Abnormality of the eye	5	0.0	0.584
Abnormality of the digestive system	34	20.6	0.81
Abnormality of metabolism/homeostasis	12	13.8	0.702
Abnormality of the genitourinary system	23	13.0	0.574
Abnormality of the nervous system	38	22.0	0.244
Abnormality of the respiratory system	27	29.6	0.18
Abnormality of the immune system	5	20.0	1
Abnormality of blood and blood-forming tissues	8	37.5	0.179
Abnormality of the integument	15	40.0	0.042
Abnormality of the ear	4	25.0	0.575
Abnormality of the endocrine system	2	100.0	0.035

The phenotypes associated with a higher diagnostic yield were “Abnormality of Prenatal Development or Birth” (30%), “Abnormality of the Genitourinary System” (30%), “Abnormality of the Musculoskeletal System” (25%) and “Abnormality of the Head or Neck” (25%). However, the number of patients (*n*) varied across each group. A statistically significant individual association (*p* ≤ 0.05) was only observed in the categories “Abnormality of the Integument” (*p*-value 0.042) and “Abnormality of the Endocrine System” (*p*-value 0.035). In contrast, this approach did not reveal discernible patterns in the phenotypic characteristics of the other categories, particularly considering that most patients exhibit multiple phenotypic traits.

**Figure 7:**
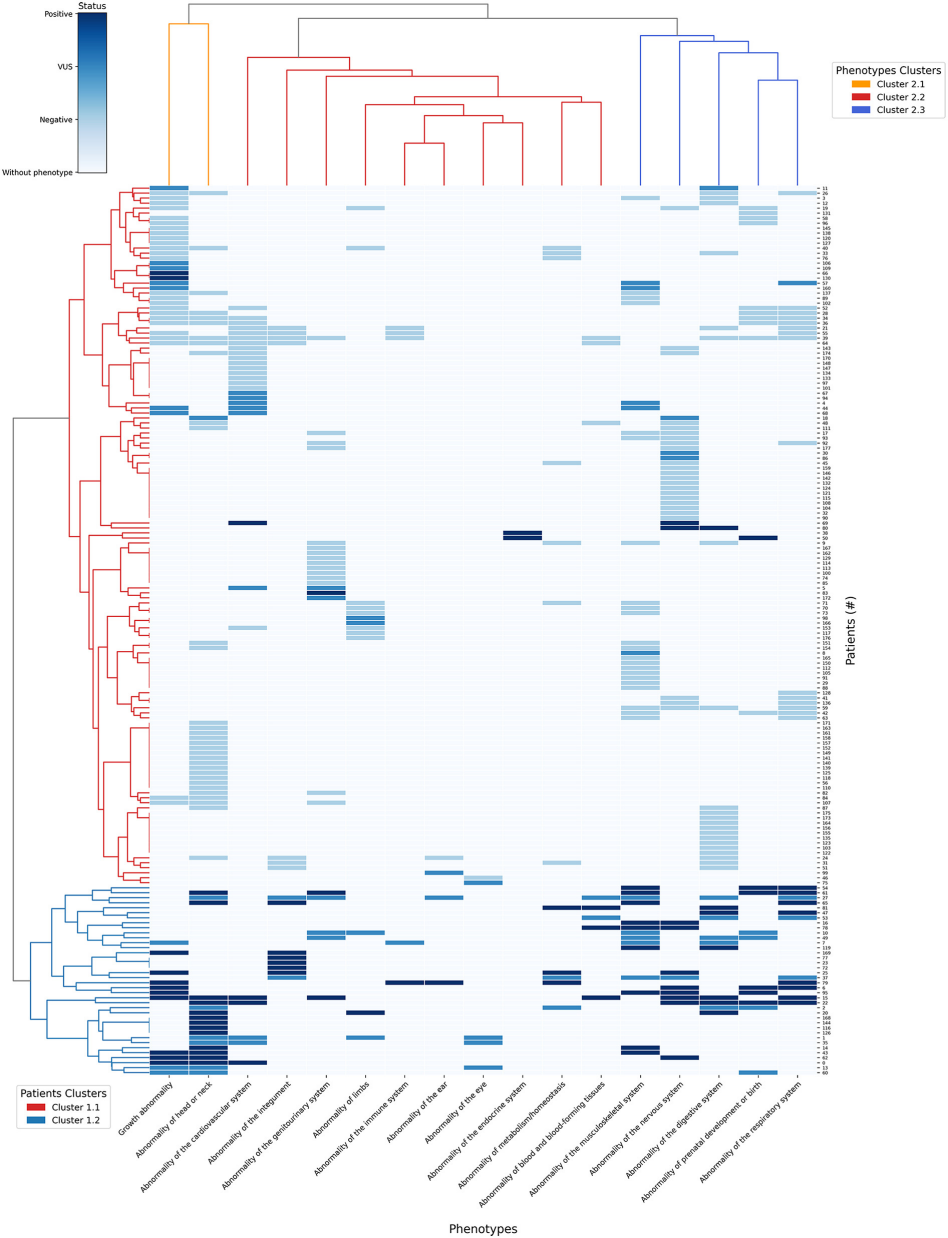
Heatmap of clustering patterns between patients and HPO terms with dendrograms.

To conduct a global analysis, we applied a clustering model to patients (rows) and associated phenotypes (columns), visualized in a heatmap with dendrograms (Figure 7). This hierarchical representation facilitated the identification of phenotypic subgroups and their correlation with genetic test results. To minimize bias, we excluded patients with incomplete phenotypic descriptions (*n* = 7), those reporting only family history (*n* = 2), and duplicate tests in patients undergoing both aCGH and WES (*n* = 18), resulting in a final cohort of 178 patients.

The heatmap employs four distinct colors: white for phenotypes absent in the patient; light blue for phenotypes present in patients with negative results; blue for phenotypes with VUS result and dark blue for phenotypes with positive results. The rows (vertical axis) represent the 178 patients and columns (horizontal axis) correspond to the 17 HPO terms where the phenotypes were grouped after propagation.

The first dendrogram (Patients Clusters) classified 178 patients in two main clusters: In cluster 1.1 (red row cluster) (*n* = 38), 27 patients were positive and 11 patients had VUS results; In Cluster 1.2 (blue row cluster) (*n* = 140), 7 patients were positive, 20 had VUS results and 113 had negative results. Cluster 1.2 exhibited a significantly higher ratio of positive results to VUS compared to cluster 1.1, suggesting a stronger association with a confirmed genetic diagnostic. Therefore, cluster 1.2 was prioritized for phenotypic analysis, as its higher diagnostic yield provided a more informative genetic profile. The distribution of phenotypes in cluster 1.2 is described on Table 4:

**Table 4:** Distribution of phenotypes in Group 1.2 of dendrogram.

Phenotype	Result	*n*
Growth abnormality	Positive	9
	VUS	3
Abnormality of head or neck	Positive	13
	VUS	6
Abnormality of the cardiovascular system	Positive	3
	VUS	2
Abnormality of the integument	Positive	6
	VUS	2
Abnormality of the genitourinary system	Positive	2
	VUS	3
Abnormality of limbs	Positive	1
	VUS	2
Abnormality of the immune system	Positive	1
	VUS	1
Abnormality of the ear	Positive	1
	VUS	1
Abnormality of the eye	Positive	0
	VUS	3
Abnormality of metabolism/homeostasis	Positive	3
	VUS	2
Abnormality of blood and blood-forming tissues	Positive	3
	VUS	2
Abnormality of musculoskeletal system	Positive	9
	VUS	5
Abnormality of the nervous system	Positive	8
	VUS	1
Abnormality of the digestive system	Positive	6
	VUS	4
Abnormality of prenatal development of birth	Positive	5
	VUS	4
Abnormality of the respiratory system	Positive	8
	VUS	3

The most common phenotype in the group of patients of Table 3 was “Abnormality of head or neck” (*n* = 19) which also had the highest number of positive results (*n* = 13). However, the phenotype with the highest proportion of positive results ratio was “Abnormality of the nervous system”, with 88% of positive results (*n* = 8 out of 9 cases). Only “Abnormality of the endocrine system” was not present in any of the 38 patients in this cluster.

In the dendrogram that grouped the 16 phenotypes (Phenotypes Clusters) there are 3 main clusters: In cluster 2.1 (orange column cluster) (*n* = 2), from 82 patients that had at least 1 of the 2 phenotypes of this cluster, 20 patients were positive, 15 had VUS results and 47 patients had negative results. In cluster 2.2 (red column cluster) (*n* = 10) from 85 patients that had at least 1 of the 10 related phenotypes, 18 were positive, 21 had VUS results and 46 had negative results. Finally for cluster 2.3 (blue column cluster) (*n* = 5), from 108 patients that have at least 1 of the 5 phenotypes in this cluster, 21 were positive, 17 had VUS results and 70 had negative results.

To compare the results of the three phenotype clusters, the diagnostic yield of each was calculated (Table 5). Taking into account that each cluster has a different number of patients and phenotypes, the diagnostic yield result was normalized in order to have a comparable proportional value.

**Table 5:** Diagnostic yield for each phenotype clusters.

Cluster	Diagnostic yield (%)	Normalized diagnostic yield (%)
2.1	24.4	12.2
2.2	21.2	2.1
2.3	19.4	3.9

Additionally, odds ratios (OR) were calculated for each cluster with a 95% confidence intervals (CI) to assess the probability of obtaining a positive result if one of the cluster phenotypes was present with either aCGH or exome. For cluster 2.1 the OR was 1.89 (95% CI: 0.88–4.03). For cluster 2.2 the OR was 0.61 (95% CI: 0.31–1.18). For cluster 2.3 the OR was 1.06 (95% CI: 0.49–2.28).

## 4 Discussion

WES detected abnormal findings (P/LP/VUS) in 53% of cases, with a diagnostic yield of 30.5%, consistent with reported rates of 21%–57% (24, 25). In contrast, aCGH identified abnormalities in 17.6% of cases, with a diagnostic yield of 8%, slightly lower than the reported 14%–34% (21, 26). Notably, in two cases, WES identified a CNV responsible for the phenotype, with aCGH used only for confirmation and refinement of genomic coordinates. These findings underscore the superior performance of WES over aCGH and highlight NGS as a valuable tool for detecting genetic causes beyond SNVs, despite not being the gold standard for CNV identification.

From a clinical perspective, genetic testing proved highly valuable for both patients and their families. In several cases, the identification of a pathogenic or likely pathogenic variant established a definitive diagnosis that informed prognosis and guided modifications in clinical management, including surveillance and supportive care. Moreover, molecular diagnoses enabled personalized genetic counseling, particularly regarding recurrence risk in families of deceased neonates. The availability of a priority post-test counseling program for all patients ensured timely and appropriate clinical support.

Fisher's exact test identified statistical significance for diagnostic yield only in “Abnormality of the integument” (*p* = 0.042) and “Abnormality of the endocrine system” (*p* = 0.035). These findings suggest a higher diagnostic yield for these phenotypes (40% and 100%, respectively). However, sample size must be considered: “Abnormality of the endocrine system” included only two patients, raising the possibility of an outlier effect, while “Abnormality of the integument” involved 15 patients, providing a stronger basis for this association, consistent with Zhu et al. (27). In contrast, other phenotypes showed yields between 0% and 37.5% but lacked statistical significance (*p* > 0.05), highlighting the need for larger samples to validate these trends.

Clustering analysis (Figure 6) minimized sample size bias and revealed phenotypic trends linked to higher diagnostic yield. In the cluster with the most positive results (cluster 1.2), “Abnormality of head or neck” was most frequent (*n* = 19) and yielded the highest number of positive cases (*n* = 13), while “Abnormality of the nervous system” exhibited the highest positivity rate (88%, 8/9 cases). Conversely, the absence of “Abnormality of the endocrine system” in this cluster (0/38) suggests these alterations may occur in different clinical contexts.

Further analysis of phenotype clusters revealed that cluster 2.1 (2 phenotypes) had a higher likelihood of a positive genomic result (OR = 1.89, 95% CI: 0.88–4.03) compared to patients lacking these features, partially supporting findings by Scholz et al. (25). In contrast, cluster 2.2 (10 phenotypes) was associated with a lower probability (OR = 0.61, 95% CI: 0.31–1.18), while cluster 2.3 (5 phenotypes) showed an intermediate association (OR = 1.06, 95% CI: 0.49–2.28). Although none reached statistical significance, these trends are biologically suggestive.

Additionally, taking into account that these are costs covered by the Colombian health system, and that the proper management of resources is essential, our results suggest that the WES should be considered over aCGH as a first-level molecular test in patients mainly with growth abnormalities and/or abnormalities of the head or neck. associated with prenatal malformations and postnatal respiratory alterations. Abnormalities of nervous, skeletal and digestive system development should also be considered.

One of the main limitations of this study is that neither patient selection nor the decision to order genetic testing was under our direct control, which restricted our ability to evaluate factors influencing the choice between WES and aCGH. This limitation reflects the broader absence of standardized guidelines for genetic testing in NICU settings in Colombia. Taking this into account, our findings underscore the need to strengthen the role of clinical genetics in neonatal intensive care and to promote the integration of genomic testing in the diagnostic evaluation of selected critically ill neonates in our country. Moreover, expanding the cohort size will be essential to enhance the statistical power of phenotype–genotype associations and to validate the trends observed in this study.

## 5 Conclusions

The diagnostic yield of WES was significantly higher than that of aCGH, with WES achieving a 30.5% diagnostic yield compared to 8% for aCGH. These results align with international studies, reinforcing the utility of these technologies in diagnosing genetic anomalies in the NICU.

Furthermore, the integration of molecular testing in neonatal care not only allows a more accurate and timely diagnosis, but also contributes to optimize the use of resources in the health system. As these tests are covered by the Colombian health system, our findings support the adoption of precision medicine strategies in the NICU, fostering early and personalized interventions that can enhance patient outcomes.

On the other hand, the propagation of HPO terms by phenotypic analysis performed in this study allowed us to identify Growth abnormality and/or Abnormality of head or neck phenotypes as those of greatest interest when considering profiling a NICU patient to a WES or aCGH molecular test to determine their diagnosis.

Although the study provides valuable evidence, its findings are limited by the cohort size, and further prospective studies are needed to validate and refine these results, considering the statistical constraints. The phenotypic heterogeneity and limited sample size in some subgroups suggest that future studies should consider patient selection and grouping strategies that allow a more robust assessment of genotype-phenotype associations.

## Data Availability

The original contributions presented in the study are included in the article/Supplementary Material, further inquiries can be directed to the corresponding author.
