# Supplementary material for: Genetic testing enhances diagnosis in critically ill neonates: insights from the first Colombian cohort
Source: Front Pediatr. 2025 Aug 21;13:1605166. doi: 10.3389/fped.2025.1605166 (PMC12408507; doi:10.3389/fped.2025.1605166)
Supplement: Supplementary file 1 [file Datasheet1.pdf]

CARE case report guidelines

CARE Checklist of information to include when writing a case report

(cc) BY-NC-ND

Topic	Item	Checklist item description	Reported on Line
Title	1	The diagnosis or intervention of primary focus followed by the words "case report"	N/A
Key Words	2	2 to 5 key words that identify diagnoses or interventions in this case report, including "case report"	N/A
Abstract (no references)	3a	Introduction: What is unique about this case and what does it add to the scientific literature?	Abstract
	3b	Main symptoms and/or important clinical findings	Abstract
	3c	The main diagnoses, therapeutic interventions, and outcomes	Abstract
	3d	Conclusion—What is the main "take-away" lesson(s) from this case?	Abstract
Introduction	4	One or two paragraphs summarizing why this case is unique (**may include references**)	1. Introduction
Patient Information	5a	De-identified patient specific information.	3.2.1. WES results
	5b	Primary concerns and symptoms of the patient.	3.2.1. WES results
	5c	Medical, family, and psycho-social history including relevant genetic information	3.2.1. WES results
	5d	Relevant past interventions with outcomes	3.2.1. WES results
Clinical Findings	6	Describe significant physical examination (PE) and important clinical findings.	3.2.1. WES results
Timeline	7	Historical and current information from this episode of care organized as a timeline	3.2.1. WES results
Diagnostic Assessment	8a	Diagnostic testing (such as PE, laboratory testing, imaging, surveys).	3.2.1. WES results
	8b	Diagnostic challenges (such as access to testing, financial, or cultural)	4. Discussion
	8c	Diagnosis (including other diagnoses considered)	3.2.1. WES results
	8d	Prognosis (such as staging in oncology) where applicable	3.2.1. WES results
Therapeutic Intervention	9a	Types of therapeutic intervention (such as pharmacologic, surgical, preventive, self-care)	N/A
	9b	Administration of therapeutic intervention (such as dosage, strength, duration)	N/A
	9c	Changes in therapeutic intervention (with rationale)	N/A
Follow-up and Outcomes	10a	Clinician and patient-assessed outcomes (if available)	N/A
	10b	Important follow-up diagnostic and other test results	3.2.1. WES results
	10c	Intervention adherence and tolerability (How was this assessed?)	N/A
	10d	Adverse and unanticipated events	3.2.1. WES results
Discussion	11a	A scientific discussion of the strengths AND limitations associated with this case report	4. Discussion
	11b	Discussion of the relevant medical literature **with references**.	4. Discussion
	11c	The scientific rationale for any conclusions (including assessment of possible causes)	N/A
	11d	The primary "take-away" lessons of this case report (without references) in a one paragraph conclusion	N/A
Patient Perspective	12	The patient should share their perspective in one to two paragraphs on the treatment(s) they received	N/A
Informed Consent	13	Did the patient give informed consent? Please provide if requested	Yes ☒ No ☐
